# Cost-effectiveness modelling of telehealth for patients with raised cardiovascular disease risk: evidence from a cohort simulation conducted alongside the Healthlines randomised controlled trial

**DOI:** 10.1136/bmjopen-2016-012355

**Published:** 2016-09-23

**Authors:** Padraig Dixon, Sandra Hollinghurst, Roberta Ara, Louisa Edwards, Alexis Foster, Chris Salisbury

**Affiliations:** 1 Centre for Academic Primary Care, School of Social and Community Medicine, University of Bristol, Bristol, UK; 2 ScHARR, The University of Sheffield, Sheffield, UK

**Keywords:** HEALTH ECONOMICS, STROKE MEDICINE, Angina

## Abstract

**Objectives:**

To investigate the long-term cost-effectiveness (measured as the ratio of incremental NHS cost to incremental quality-adjusted life years) of a telehealth intervention for patients with raised cardiovascular disease (CVD) risk.

**Design:**

A cohort simulation model developed as part of the economic evaluation conducted alongside the Healthlines randomised controlled trial.

**Setting:**

Patients recruited through primary care, and intervention delivered via telehealth service.

**Participants:**

Participants with a 10-year CVD risk ≥20%, as measured by the QRISK2 algorithm, and with at least 1 modifiable risk factor, individually randomised from 42 general practices in England.

**Intervention:**

A telehealth service delivered over a 12-month period. The intervention involved a series of responsive, theory-led encounters between patients and trained health information advisors who provided access to information resources and supported medication adherence and coordination of care.

**Primary and secondary outcome measures:**

Cost-effectiveness measured by net monetary benefit over the simulated lifetime of trial participants from a UK National Health Service perspective.

**Results:**

The probability that the intervention was cost-effective depended on the duration of the effect of the intervention. The intervention was cost-effective with high probability if effects persisted over the lifetime of intervention recipients. The probability of cost-effectiveness was lower for shorter durations of effect.

**Conclusions:**

The intervention was likely to be cost-effective under a lifetime perspective.

**Trial registration number:**

ISRCTN27508731; Results.

**Strengths and limitations of this study**

- The Healthlines trial was one of the largest randomised controlled trials designed to evaluate a telehealth-based complex intervention for the management of cardiovascular disease risk.
- This study complements and extends the findings of a within-trial evaluation carried out on outcomes at 12 months from randomisation by evaluating the cost-effectiveness of the telehealth intervention over the lifetime of trial participants using a cohort simulation model.
- The intervention is likely to be cost-effective from a health system perspective, but we cannot identify the most plausible duration of intervention effect.

## Introduction

Cardiovascular disease (CVD) is a prevalent long-term condition associated with substantial morbidity and mortality.^1^ Effective care for patients with raised CVD risk requires attention to, and management of, underlying risk factors such as high blood pressure (BP) and obesity.^2^ A challenge in estimating the cost-effectiveness of interventions intended to reduce CVD risk is that randomised controlled trials (RCTs) may have follow-up periods that are shorter than the period over which an intervention may affect CVD risk. This is in spite of the impact that interventions to reduce CVD risk—such as BP management and weight management programmes—might have in reducing the future occurrence of debilitating events such as acute myocardial infarction (AMI) and stroke.

Telehealth is one form of condition management that may be relevant to long-term conditions in general and to CVD in particular.^3^ However, there is a lack of high-quality evidence regarding the cost-effectiveness of telehealth.^4–7^ To estimate the clinical and cost-effectiveness of a de novo telehealth intervention, the Healthlines CVD risk RCT individually randomised 641 participants to receive either usual care or a theory-based telehealth intervention that encouraged participants to engage in beneficial behaviour change. The primary clinical outcome of the trial was 10-year CVD risk, measured using the QRISK2 risk prediction algorithm^8^ at the end of 12 months of follow-up.

In the absence of data from long-term follow-up, there is no means of assessing the enduring impact on cost-effectiveness of the changes in modifiable risk factors that the intervention may have wrought. For example, it is plausible that some patients who managed to reduce their BP, body mass index (BMI) or whose medication adherence was improved as a result of the intervention may continue with newly acquired beneficial behaviours after the end of the 12 months of trial follow-up. Indeed, it is implausible that all trial participants would immediately revert to their preintervention BP or BMI immediately on trial completion.

This paper describes the development and analysis of a state transition cohort simulation cost-effectiveness model intended to estimate the expected net benefits of the Healthlines telehealth intervention over the lifetime of trial participants. This approach is consistent with the principles of economic evaluation,^9^ guidance from the National Institute for Health and Care Excellence (NICE)^10^ and ISPOR guidelines^11^ which recommend that cost-effectiveness analyses be undertaken over a time period that reflects differences in cost and effect attributable to an intervention or treatment. The long-term cost-effectiveness analysis complements the analysis of cost-effectiveness of the intervention at 12 months postrandomisation published in a companion paper.^12^

### The Healthlines RCT

The trial protocol, methods, results and cost-effectiveness analyses have been reported elsewhere.^3^ ^13^ Briefly, patients aged between 40 and 74 with 10-year CVD risk ≥20% (measured by the QRISK2 algorithm) and at least one modifiable risk factor (eg, weight management or smoking) were recruited from 42 GP practices in England. Individuals were not considered if they had a confirmed diagnosis of CVD, were pregnant, did not have access to the internet and telephones or were unable to communicate verbally. More detail on these and other exclusion criteria is available in Salisbury *et al*.^3^ In total, 641 patients were randomised, and received either unmodified usual care (n=316) or a telehealth intervention in addition to usual care (n=325), for a follow-up period of 12 months.

Participants in the intervention arm could receive up to 13 responsive, tailored telehealth encounters with trained Health Information Advisors. Participants were encouraged to access and use online material and apps related to CVD risk. Participants with systolic BP ≥140 mm Hg and without atrial fibrillation were offered a BP monitor to use at home, and could upload readings to a portal to monitor progress. Telephone calls focused on goal setting, stimulus control and the management of CVD risk in a way that was relevant to the circumstances of each individual participant and which was in line with NICE guidelines. This included ensuring that patients were taking appropriate drug treatment, and addressing problems with medication adherence.

The within-trial economic evaluation at the end of 12 months of follow-up adopted an NHS perspective. The cost-effectiveness of the intervention was estimated against NICE thresholds using NHS cost data collected from medical records and questionnaires during the trial, and using data on quality-adjusted life years (QALYs) estimated from responses to the EQ-5D-5L^14^ generic quality-of-life measure at baseline, and 6 and 12 months from randomisation. The mean incremental NHS costs in 2012/2013 prices were estimated to be £138 per patient (ie, the intervention was more expensive), and the mean incremental QALY gains were estimated to be 0.012. The estimated ICER was £10 859, and the estimated net benefit at a threshold of £20 000 was estimated to be £116 (95% CI: −£58 to £291). The probability that the intervention was cost-effective at this threshold value was 0.77.

## Methods

### Model structure

**Figure 1:**
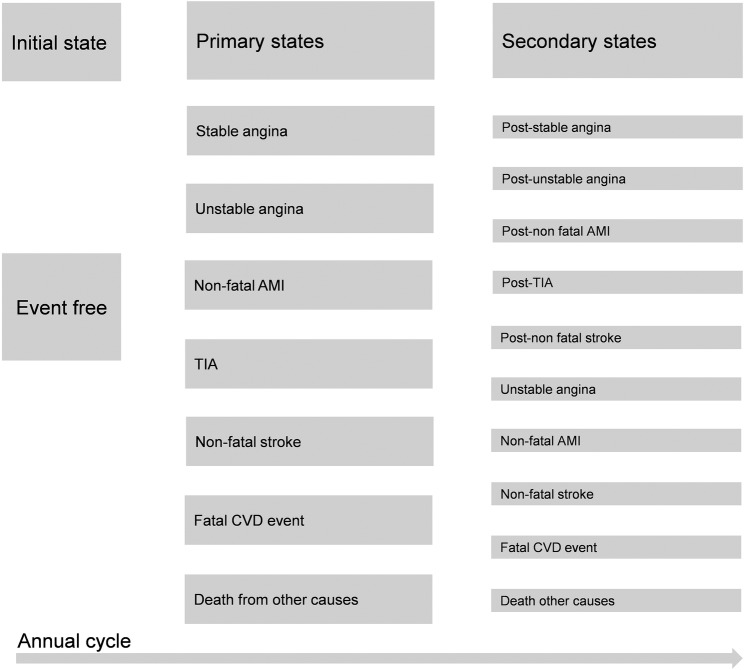
Simulated model states. This figure is based on Figure 27 of Ward *et al*.^24^

Cohorts of 1000 patients were simulated. These cohorts were based on the characteristics of the 641 trial participants. Cohorts differed according to age at randomisation, sex and trial allocation of the patient groups modelled. A set of discrete, mutually exclusive states were created and defined by the presence (or absence) of a CVD-related condition, or with death (figure 1). Sequences of states experienced by the simulated cohort members defined clinical pathways in which QALYs (associated with utilities and mortality) and costs were accumulated. Cost-effectiveness, as measured by the averages of costs and QALYs in each arm, was the primary outcome. These averages were calculated and allowed for the construction of net benefit statistics. Costs were expressed in 2012/2013 sterling prices.

The model is based on adapted updated versions of models used in previous health technology assessments in the area of CVD risk.^15^ ^16^ A National Health System perspective was adopted. The model cycled annually. Costs and outcomes were subject to a half-cycle correction.^17^ Consistent with the inclusion criteria of the trial, and the construction of the QRISK2 algorithm,^8^ patients were assumed to be free of CVD when they entered the model—this corresponds to the ‘event free’ state. Subsequent states correspond to the occurrence of the events used in the QRISK2 algorithm—AMI, angina, transient ischaemic attack (TIA) and stroke—and to postevent states, for example, a ‘poststroke’ state (figure 1).

Patients cycled through the model until death, or age 100, at which point they were assumed to die. A total of 1000 iterations of the model were simulated, in which values of input parameters were varied simultaneously. Each iteration used a value drawn from probability distributions assigned to particular input parameters of the model as described below, in the online supplementary table A2 and in Salisbury *et al*.^3^ Probabilistic sensitivity analysis was used to quantify uncertainty around point estimates of net benefit. The model was implemented in Excel 2013 (Microsoft, Redmond, Washington, USA).

10.1136/bmjopen-2016-012355.supp1 supplementary appendix

### Probability of transitioning to primary and secondary states

The probability of entering a particular state is determined by CVD risk. Initial risk scores of patients at the beginning of the model, corresponding to the event-free state, were based on information collected in the RCT (table 1). CVD risk in the initial year (year 0) is thus average participant baseline QRISK2 score. Change in CVD risk at the end of this year/start of the next year (year 1) is measured by QRISK2 score calculated at 12-month follow-up and adjusted for baseline risk, details of which are provided in Salisbury *et al*.^3^ CVD risk in subsequent years was obtained by adding percentage increases obtained from growth rates in annual risk in males when all input values to the QRISK2 algorithm other than age were held constant.

**Table 1:** QRISK2 scores at baseline and after 12 months of follow-up

	Control arm		Intervention arm	
	Baseline	12-month follow-up, adjusted for baseline	Baseline	12-month follow-up, adjusted for baseline
QRISK2 score for males	31.59	32.00	31.83	31.60
QRISK2 score for females	27.73	28.24	27.86	27.84

The data are presented as levels of QRISK2 as it is the level of risk that determines the probability of events such as stroke or acute myocardial infarction.

These data are based on imputed QRISK2 scores—multiply imputed data are used in the base case economic evaluation at 12 months from randomisation.^3^ These data are based on CVD risk of all of those randomised (n=641) in the RCT. The methods used for multiple imputation are described in further detail in the companion 12-month economic evaluation paper.^12^

Growth rates from men were used because the level of CVD risk is higher for men than for women at all ages, other variables being constant. Using the relatively more rapid growth rates observed in female risk levels as a basis for extrapolation would have meant that at some ages, female risk was much higher than male risk. The use of male risk growth rates constrains female risk levels to be below male levels at all ages, ensures that risk does not rise above 100%, better reflects estimated prevalence of risk in males and females, and more generally ensures that the cost-effectiveness results are based on plausible risk profiles.^3^

The QRISK2 algorithm is not validated for ages beyond 84. Risk for patients surviving to 85 and above was obtained by extrapolating 10-year risk by 1% per annum, which corresponds to the coefficient obtained from an ordinary least squares regression of QRISK2 scores on age.

Ten-year risk scores were converted into an annual risk of any event to reflect the annual cycles of the model. Annual risk scores of *any* defined CVD event were then disaggregated into the risk of a *specific* event—AMI, angina, TIA and stroke—using data on the incidence of these conditions by age and sex. The sources for these data and the input values used are described in the online supplementary tables A5 and A6. Further detail on their calculation is available in Salisbury *et al*.^3^

These incidence rates establish the probability of moving from the event-free state to a specific CVD-related event state; that is, they establish the risk of a primary event. Transitions to secondary events (any event after the primary event) are determined by the age and sex-specific transition probabilities given in Ara *et al*,^15^ who describe in detail the data sources and methods used in their construction. Permitted transitions between states are listed in the online supplementary table A1.

The probability of death due to causes not related to CVD risk was based on standardised mortality ratios calculated from the most recently available (2012) Office of National Statistics Interim Life Tables.^18^ This mortality ratio excluded CVD-related ICD-10 codes (I20–I25 and I60–I69). The risks of mortality used in each iteration of the model were based on draws from a univariate normal distribution.

Simulated participants were constrained to experience no more than three CVD events, an assumption that reflected data availability and model tractability. The same assumption was used in Ara *et al*.^15^ However, few of the 1000 simulated participants approached experiencing three events, and therefore, the assumption is unlikely to have influenced cost-effectiveness conclusions.

### Duration of effect of intervention

The duration of the effect of the Healthlines intervention on CVD risk beyond the end of trial follow-up is unknown, but the modelling thereof is the principal motivation for undertaking the simulation modelling described here. Four different scenarios based on different durations of effect were modelled, and their implications for cost-effectiveness compared. This is similar to the approach of Mistry *et al*.^19^

The scenarios modelled are of durations of intervention effect of 1, 2 and 5 years from baseline, and for remaining lifetime. If, for example, the duration effect is 2 years, then participants in the intervention arm receive the benefit of lower risk for 2 years, after which they are modelled as having the same sex- and age-adjusted risk as participants in the control arm. Similar logic applies to the other scenarios. In the case of a lifetime duration of effect, risk is permanently lower in the intervention arm. To avoid sudden ‘jumps’ in risk at the point at which the intervention ends, the model uses a smoothing adjustment which calculates the average score of intervention arm risk and usual care risk for the year after which the modelled duration of effect expires.

### Data on utility

QALYs in year 0 of the model were obtained from participant responses to the EQ-5D-5L measure at baseline, 6 and 12 months of RCT follow-up and were ‘cross-walked’ to UK EQ-5D-3L instrument and valued using the Euroqol UK value set.^20^ QALYs were adjusted to account for between-arm differences observed at the end of trial follow-up. For other years of the model, age- and sex-adjusted population, EQ-5D norms were estimated from Ara and Brazier^21^ to provide baseline utility scores that applied to the event-free state. These EQ-5D data were used to calculate QALYs.

Multiplicative adjustments, which assume a constant proportional effect on baseline utility,^21^ were made to reflect the decremental impact of experiencing CVD-related health states. Ara and Brazier^21^ was used as the source of state-specific utilities other than TIA, which were drawn from Luengo-Fernandez *et al*,^22^ and for unstable angina. It was assumed that the utility estimates for angina in Ara and Brazier related to stable angina; it was further assumed that utility associated with the unstable angina health state would be 90% of the stable angina values, as in Ara *et al*.^15^

Condition-specific utility values were drawn from univariate normal distributions, as recommended by Ara and Wailoo,^23^ to reflect uncertainty in mean values. Utilities corresponding to postevent states were never lower than for the event state itself. This means, for example, that utilities of the ‘poststroke’ event state were higher than or equal to the ‘stroke’ state, implying that experiencing a stroke had a greater negative impact on average on patient utility than did experiencing the ‘poststroke’ state.

It was assumed that the second and third events (such as second and third non-fatal strokes) had the same impact on utility as the first event. This rules out multiplicative impacts of events on quality of life. The utility values associated with different states are presented in the online supplementary material table A3.

### Data on cost

A health-system perspective was adopted for the analysis, and hence only NHS costs were considered. This excluded consideration of any personal or societal costs associated with, for example, incapacity and exit from the labour force connected with a debilitating but non-fatal stroke.

The costs of health states were based, in most cases, on Ward *et al*^24^ and Ara *et al*.^15^ The first year of the model also included costs of the intervention, and NHS costs incurred in each arm. The costs of each health state are described in the online supplementary table A4.

The cost estimates in Ward *et al*^24^ and Ara *et al*,^15^ derived from systematic reviews, were updated where appropriate and possible with new data, as described in more detail in Salisbury *et al*.^3^ Mean costs enter the simulation model at each iteration as draws from a γ distribution.

### Cost-effectiveness analysis

The simulation model uses information on the NHS costs and QALYs observed during the 12 months of trial follow-up in the initial year (year 0). All subsequent costs and QALYs are simulated by the model as a function of QRISK2 scores using the methods described above. Costs and outcomes were discounted at 3.5% per year, in line with NICE recommendations.^10^ Cumulative costs and QALYs were obtained for the lifetime of cohort members by calculating probability-weighted averages of the number of patients in each state, the length of time patients have been in particular states, and the costs, mortality and quality of life associated with each state.

## Results

The mean age of men (women) in the trial was 67 (69), and men comprised 80% of trial participants. Table 1 contains baseline and adjusted 12-month QRISK2 scores—higher scores indicate higher CVD risk.

**Figure 2:**
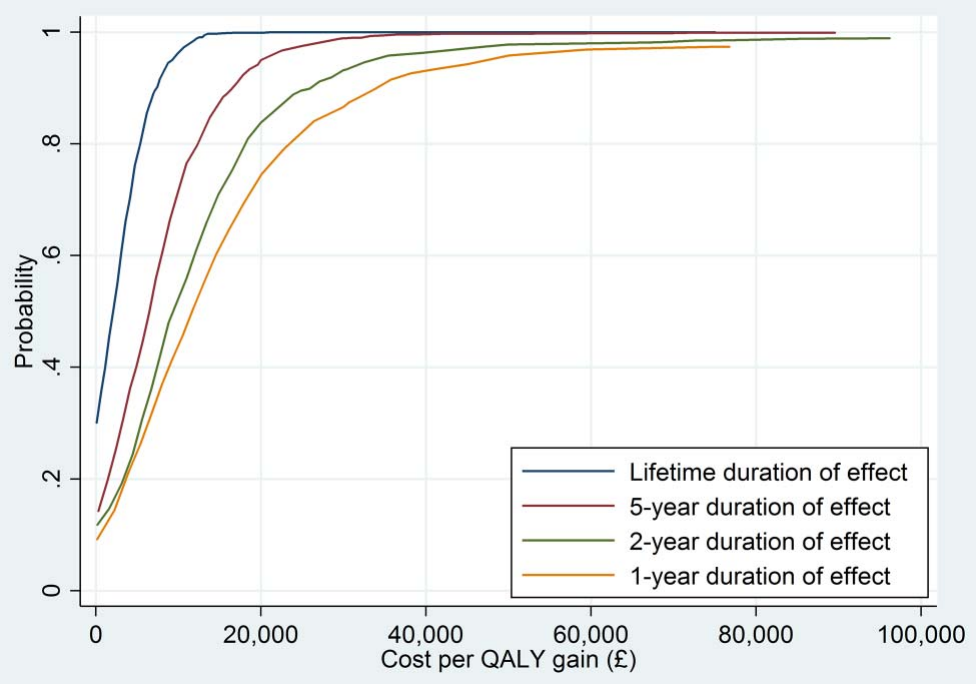
Cost-effectiveness acceptability curves from lifetime simulation assuming different durations of effect.

**Figure 3:**
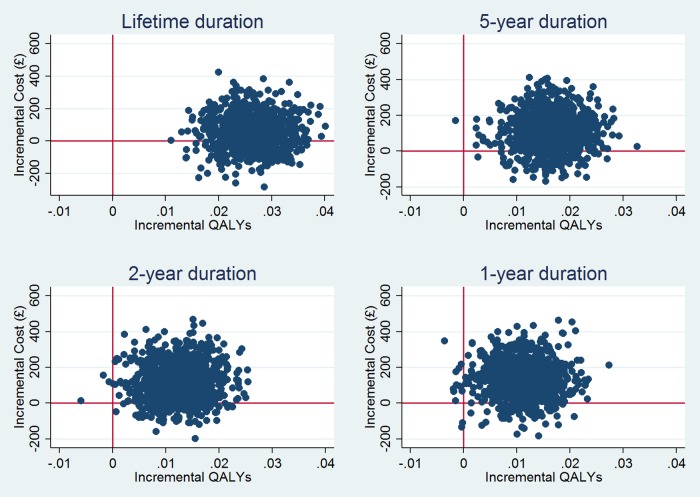
Cost-effectiveness planes from lifetime simulation assuming different durations of effect.

Table 2 provides the results of the cost-effectiveness analysis for different durations of intervention effect. The longer the assumed duration of effect, the more likely it is that the intervention is cost-effective at cost-effectiveness thresholds conventionally employed (ie, £20 000 per QALY). It is notable that, even if the beneficial effects of the intervention on CVD risk last only 1 year, the intervention is likely to be cost-effective. This is also apparent in the cost-effectiveness acceptability curves, where a monotonic relationship between duration of the probability of cost-effectiveness is evident (figure 2). The corresponding cost-effectiveness planes for each scenario illustrate this point in a different way (figure 3). At longer durations of effect, almost all of the 1000 simulated incremental cost/incremental pairs are in the northeast or southeast quadrants. At shorter durations, an increasing but still modest number of these pairs are in the northwest or southwest quadrants.

**Table 2:** Base case cost-effectiveness results for different durations of effect: per-patient average costs and effects

	Modelled duration of effect			
	1 year	2 years	5 years	Lifetime (permanent) effect
Control arm NHS costs	£6595	£6617	£6608	£6602
Intervention arm NHS costs	£6726	£6741	£6714	£6657
Control arm QALYs	8.573	8.573	8.573	8.572
Intervention arm QALYs	8.584	8.586	8.589	8.598
Incremental costs (95% CI)	£131 (£125 to 138)	£124 (£118 to 130)	£107 (101 to 112)	£55 (49 to 61)
Incremental QALYs (95% CI)	0.011 (0.011 to 0.011)	0.013 (0.012 to 0.013)	0.016 (0.016 to 0.017)	0.026 (0.026 to 0.027)
ICER	£11 776	£9886	£6477	£2091
Probability cost-effective at £20 000 threshold	0.74	0.84	0.95	0.99
Probability cost-effective at £30 000 threshold	0.87	0.93	0.99	1.00
NMB at threshold of £20 000 (95% CI)	£92 (−172 to 352)	£127 (−144 to 382)	£223 (−153 to 468)	£472 (197 to 728)

CE, cost-effectiveness; ICER, incremental cost-effectiveness ratio; NMB, net monetary benefit; QALYs, quality-adjusted life years.

The small QRISK2 difference between arms means that the control and intervention groups have very similar risk profiles once the assumed duration of effect expires. The lifetime cost-effectiveness results are therefore influenced, especially at short durations of effect, by the inclusion of costs and QALYs observed during the 12-month period of trial follow-up. The between-arm cost and QALY differences in the initial 12 months are propagated forward over the remaining lifetime of trial participants, and are more influential than the subsequent minor differences in costs and QALYs associated with different levels of simulated QRISK2. Nevertheless, the differences in QRISK2 are consequential at longer durations of effect, since they are associated with fewer CVD events in the intervention arm.

## Discussion

Cost-effectiveness over the lifetime of participants in the Healthlines telehealth trial was estimated using a cohort simulation model. The probability of cost-effectiveness increased with longer durations of effect, but even with the shortest duration of effect, the intervention was estimated to be cost-effective. These results reflect the cost-effectiveness analysis at 12 months: because of the small difference in QRISK2 between arms, the QALY difference observed at the end of trial follow-up has a major influence on lifetime cost-effectiveness.

The model is subject to a number of limitations. The quality of the output reflects the assumptions and data used to construct and populate the model. The QRISK2 algorithm is not validated for ages beyond 84, and we made an assumption as to growth in risk for the small number of simulated trial participants who lived to 85 and beyond.

As in all modelling exercises of this type, the results may be sensitive to structural assumptions, such as the number of health states modelled.^11^ We cannot eliminate the possibility that alternative representations of clinical pathways may produce different conclusions. However, we have relied on a representation of health states that is consistent with the QRISK2 algorithm, the defined events (eg, stroke) of which are represented as distinct states in the model.

The model understates uncertainty associated with QRISK2 because the variance structures underlying the algorithm are not published or otherwise available, and the consequence of this is to narrow CIs around net monetary benefit statistics. This may have implications, in particular, for the probability of cost-effectiveness.

We have not accounted for non-health service costs, which may have consequences for carers and the wider economy for some of the conditions modelled, such as stroke.^25^ However, it is not clear if accounting for these costs would necessarily alter the between-arm comparison of cost-effectiveness, since slightly fewer fatal and non-fatal strokes would be experienced in the intervention arm.

Finally, the analysis does not identify the most plausible duration of effect. Evidence from long-term follow-up is necessary to conclusively answer this question, although synthesis of evidence from similar trials and relevant observational studies may also offer a useful complementary approach to long-term follow-up.

### Comparison with other literature

Comparison of the findings of this study with other literature assessing the long-term cost-effectiveness of interventions directed at managing CVD risk is complicated by differences in the study design used to inform the modelling, patient profiles and the nature of the interventions examined. However, the approach of using different assumed durations of effect, or different effect sizes, is encountered in similar studies.

For example, the evaluations of CVD interventions from a long-term perspective undertaken by Mistry *et al*,^19^ Asaria *et al*^26^ and Penaloza-Ramos *et al*^27^ are all sensitive to some degree to assumptions made concerning the duration of intervention effect. This is in spite of differences between these studies in population characteristics, CVD risk equation used and interventions studied, which preclude a comparison of the findings of the present study and these other analyses.

Studies also differ in the study design used to inform long-term cost-effectiveness modelling. The effect estimates used as the basis of modelling in our study are those of the ‘intention to treat’ analysis of the Healthlines RCT. This is a source of difference between the present paper and, for example, the economic modelling undertaken in support of the routine use of ‘health checks’ in the NHS in England.^28^ The economic model developed to analyse the consequences of these health checks was based on a much more extensive simulation involving synthesis of various model parameters, compliance rates and effect estimates from a variety of sources.

Ultimately, although modelling and evidence synthesis will continue to be important sources of evidence in this area, definitive evidence on the long-term consequences for CVD risk of Healthlines (and similar interventions) will require RCTs with long-term follow-up.

## Conclusion

The Healthlines telehealth service is likely to be cost-effective for individuals with raised CVD risk when a lifetime perspective is adopted. Results are somewhat sensitive to the assumed duration of intervention effect. When considering the deployment of large-scale telehealth programmes in this area, decision makers may need to consider the longevity of the behavioural and other changes rendered by the type of telehealth service studied in the Healthlines RCT, alongside other evidence concerning the long-term cost-effectiveness of similar interventions for CVD risk.
