# Supplementary material for: Cost-effectiveness modelling of telehealth for patients with raised cardiovascular disease risk: evidence from a cohort simulation conducted alongside the Healthlines randomised controlled trial
Source: BMJ Open. 2016 Sep 23;6(9):e012355. doi: 10.1136/bmjopen-2016-012355 (PMC5051382; doi:10.1136/bmjopen-2016-012355)
Supplement: supplementary appendix [file bmjopen-2016-012355supp_appendix.pdf]

Appendix – online supplementary information

A1 Permitted transitions in simulation model

Event free state to primary states

Event free to Primary Stable Angina (SA)

Event free to Primary Unstable Angina (USA)

Event free to Primary AMI

Event free to Primary TIA

Event free to Primary Stroke

Event free to Fatal CVD event (FCVD)

Event free to Death other causes (DOC)

Primary states to subsequent states

PRIMARY_SA to SECONDARY_(USA or 1st AMI or 1st Stroke or FCVD or DOC) else post SA

PRIMARY_USA to SECONDARY_(1st AMI or 1st Stroke or FCVD or DOC) else post USA

PRIMARY_AMI to SECONDARY_(1st AMI or 1st Stroke or FCVD or DOC) else post AMI

PRIMARY_TIA to SECONDARY_(1st AMI or 1st Stroke or FCVD or DOC) else post TIA

PRIMARY_Stroke to SECONDARY_(1st Stroke or FCVD or DOC) else post Stroke

Post-primary states to subsequent states

Post PRIMARY_SA to SECONDARY_(USA or 1st AMI or 1st Stroke or FCVD or DOC) else post SA

Post PRIMARY_USA to SECONDARY_(1st AMI or 1st Stroke or FCVD or DOC) else post USA

Post PRIMARY_AMI to SECONDARY_(1st AMI or 1st Stroke or FCVD or DOC) else post AMI

Post PRIMARY_TIA to SECONDARY_(1st AMI or 1st Stroke or FCVD or DOC) else post TIA

Post PRIMARY_Stroke to SECONDARY_(1st Stroke or FCVD or DOC) else post Stroke

Secondary state transitions

SECONDARY_SA to SECONDARY_((USA or 2nd AMI or 2nd Stroke or FCVD or DOC) else post SA

SECONDARY_USA to SECONDARY_((2nd AMI or 2nd Stroke or FCVD or DOC) else post USA

SECONDARY_AMI to SECONDARY_((2nd AMI or 2nd Stroke or FCVD or DOC) else post AMI

SECONDARY_TIA to SECONDARY_((2nd AMI or 2nd Stroke or FCVD or DOC) else post TIA

SECONDARY_Stroke to SECONDARY_((2nd Stroke or FCVD or DOC) else post Stroke

Post SECONDARY_SA to SECONDARY_(USA or 2nd AMI or 2nd Stroke or FCVD or DOC) else post SA

Post SECONDARY_USA to SECONDARY_(2nd AMI or 2nd Stroke or FCVD or DOC) else post USA

Post SECONDARY_AMI to SECONDARY_(2nd AMI or 2nd Stroke or FCVD or DOC) else post AMI

Post SECONDARY_TIA to SECONDARY_(2nd AMI or 2nd Stroke or FCVD or DOC) else post TIA

Post SECONDARY_Stroke to SECONDARY_(2nd Stroke or FCVD or DOC) else post Stroke

Notes to A1: SA = Stable angina; USA = Unstable angina; AMI = Acute myocardial infarction; TIA = Transient ischaemic attack; FCVD = Fatal cardiovascular disease event; DOC = Death other causes
This is the same list of permitted transitions described as in Ara et al [1] and Ara et al [2]. A1 contains the same information as Table 82 in Ara et al [1].

INPUT VALUES, REFERENCES AND PROBABILITY DISTRIBUTIONS FOR MODEL PARAMETERS

Table A2 provides a map to the various model parameters, input values, and associated probability distributions from which input values for specific model runs were drawn, and which are presented in more detail where applicable.

A2 Key input parameters and associated probability distributions

Parameter	Input values	Probability distribution	Comments
**Health state utility values**	Various; listed in Table A3	Normal	Ara and Wailoo[3] recommend the use of normal distributions to characterise the uncertainty in mean utilities
**Health state costs**	Various, listed in Table A4	Gamma	A number of sources, eg Gray et al [4], recommend using gamma distributions in characterising uncertainty in cost variables
**Incidence rates for primary CVD events**	Various, listed in Table A5 and Table A6	N/A	We follow Ward et al[5] and Ara et al[2] in using the mean values of published incidence rates in each model run
**CVD Risk**	Based on age and sex adjusted QRISK2 scores from baseline and 12m follow-up that are	N/A	The variance structures underlying the QRISK2 algorithm are not published and hence we

	presented in Table 1 in the main text, and then adjusted subsequently using the QRISK2 algorithm		could not characterise uncertainty in this measure. See Discussion in main paper.
**Risk of death from non-CVD causes**	Based on standardised mortality ratios published by the ONS	Normal distribution	We follow Ara et al[2] in using a normal distribution for model draws

UTILITIES

Ara and Brazier[6] was used as the source of state-specific utilities other than TIA, which are based on Luengo-Fernandez et al[7]. It was assumed that the utility estimates for angina in Ara and Brazier related to stable angina, and that unstable angina health utilities would be 90% of the stable angina values, as in Ara et al.[2]

As further noted in the main text, any ‘third’ events, i.e. those occurring after a secondary event, have the same utility as the ‘second’ event.

A3 Health state utility values

Health state	Mean utility value
Primary stable angina	0.615
Primary unstable angina	0.556
Primary AMI	0.721
Primary TIA	0.760
Primary stroke	0.626
Post-primary angina	0.775
Post-primary unstable angina	0.701
Post-primary AMI	0.742
Post-primary TIA	0.78
Post-primary stroke	0.668
Secondary stable angina	0.541
Secondary unstable angina	0.489
Secondary AMI	0.431
Secondary TIA	0.760
Secondary stroke	0.479
Post-secondary stable angina	0.715
Post-secondary unstable angina	0.647
Post-secondary AMI	0.685
Post-secondary TIA	0.78
Post-secondary stroke	0.641

Costs

A detailed discussion of the cost data used to model each state is provided in Salisbury et al.[8] Briefly, the costs of stable and unstable angina, of non-fatal acute myocardial infarction a are based on Ward et al[5] and Ara et al.[2] The cost of fatal

acute myocardial infarction are based on Clarke et al.[9] The costs of non-fatal stroke and of transient ischaemic attack are based on Luengo-Fernadez et al.[7] The cost of fatal stroke is based on Youman et al.[10] In all cases, costs were adjusted to 2012/13 sterling prices as described in Salisbury et al.[8]

A4 Health state costs

Health state	Mean cost in 2012/13 £ prices
Stable angina	606
Stable angina in subsequent years	356
Unstable angina	4,324
Unstable angina in subsequent years	453
Non-fatal acute myocardial infarction	3,362
Post non-fatal acute myocardial infarction	356
Fatal acute myocardial infarction	1,846
Transient ischaemic attack	3,963
Transient ischaemic attack in subsequent years	1,380
Non-fatal stroke	8,989
Non-fatal stroke in subsequent years	1,976
Fatal stroke	9,493

INCIDENCE RATES FOR PRIMARY EVENTS

Incidence rates for primary events are used to calculate the probability of one of stable angina, unstable angina, acute myocardial infarction (AMI), stroke, and transient ischaemic attack (TIA). These incidence rates are then used to disaggregate the risk of any such event, measured by the QRISK2 score, into the risk of a single event.

The data sources and adjustments made to data are described in more detail in Salisbury et al. [8] Here, we briefly describe these sources, and present in A5 and A6 the incidence data used in the simulation model.

The incidence of angina was taken from data for England reported British Heart Foundation Coronary Heart Disease Statistics 2012,[11] using the data reported for England. This was split into incidences of stable and unstable angina using incidences reported in Sutcliffe et al. [12] Incidence of fatal and non-fatal AMI was also taken from British Heart Foundation Coronary Heart Disease Statistics 2012.[11] Incidence of first ever stroke was based on Wang et al, [13] and incidence on mortality from strokes was based on Lee et al. [14]

Incidence of TIA is taken from British Heart Foundation Stroke Statistics 2009. [15]

A5 Incidence rates of CVD events per 1,000 males per year

Age band	Stable angina	Unstable angina	Non-fatal AMI	Fatal AMI	TIA	Non-fatal stroke	Fatal stroke
55-64	0.84	0.25	0.28	0.04	0.74	0.65	0.08
65-74	1.16	0.35	0.42	0.11	1.45	1.27	0.16
75-84	0.75	0.23	0.73	0.29	3.27	2.73	0.35
85+	0.75	0.23	1.23	0.76	7.94	5.12	0.65

A6 Incidence rates of CVD events per 1,000 females per year

Age band	Stable angina	Unstable angina	Non-fatal AMI	Fatal AMI	TIA	Non-fatal stroke	Fatal stroke
55-64	0.35	0.11	0.07	0.02	1.05	0.63	0.14
65-74	0.74	0.22	0.18	0.06	2.18	1.23	0.28
75-84	0.57	0.17	0.38	0.22	5.61	2.64	0.6
85+	0.57	0.17	0.75	0.64	9.14	4.95	1.13

References

1. Ara R, Tumur I, Pandor A, et al. Ezetimibe for the treatment of hypercholesterolaemia. In: NICE, ed. Technology Assessment report: The University of Sheffield, School of Health and Related Research (ScHARR), 2006.
2. Ara R, Tumur I, Pandor A, et al. Ezetimibe for the treatment of hypercholesterolaemia: a systematic review and economic evaluation. Health Technol Assess 2008;**12**(21):iii, xi-xiii, 1-212.
3. Ara R, Wailoo A. Using Health State Utility Values in Models Exploring the Cost-Effectiveness of Health Technologies. Value Health 2012;**15**(6):971-74.
4. Gray A, Clarke PM, Wolstenholme JL, et al. *Applied Methods of Cost-effectiveness Analysis in Healthcare*. Oxford: Oxford University Press, 2010.
5. Ward S, Lloyd Jones M, Pandor A, et al. A systematic review and economic evaluation of statins for the prevention of coronary events. Health Technol Assess 2007;**11**(14):178.
6. Ara R, Brazier JE. Populating an Economic Model with Health State Utility Values: Moving toward Better Practice. Value Health 2010;**13**(5):509-18.
7. Luengo-Fernandez R, Gray AM, Bull L, et al. Quality of life after TIA and stroke: Ten-year results of the Oxford Vascular Study. Neurology 2013;**81**(18):1588-95.
8. Salisbury C, O'Cathain A, Thomas C, et al. Telehealth for patients with long-term health conditions: development and evaluation of the Healthlines Service. Health Technol Assess Under Review.
9. Clarke P, Gray A, Legood R, et al. The impact of diabetes-related complications on healthcare costs: results from the United Kingdom Prospective Diabetes Study (UKPDS Study No. 65). Diabet Med 2003;**20**(6):442-50.
10. Youman P, Wilson K, Harraf F, et al. The Economic Burden of Stroke in the United Kingdom. Pharmacoeconomics 2003;**21**(5):43-50.
11. Townsend N, Wickramasinghe K, Bhatnagar P, et al. Coronary heart disease statistics 2012 edition. London: British Heart Foundation, 2012.
12. Sutcliffe S, F FK, A. WD, et al. Incidence of coronary heart disease in a health authority in London: review of a community register. BMJ 2003;**326**(7379):20.
13. Wang Y, Rudd AG, Wolfe CDA. Age and Ethnic Disparities in Incidence of Stroke Over Time: The South London Stroke Register. Stroke 2013;**44**(12):3298-304.
14. Lee S, Shafe ACE, Cowie MR. UK stroke incidence, mortality and cardiovascular risk management 1999–2008: time-trend analysis from the General Practice Research Database. BMJ Open 2011;**1**(2).
15. Scarborough P., Peto V., Bhatnagar P., et al. British Heart Foundation Stroke statistics 2009, 2009.
